# A uniform cloning platform for mycobacterial genetics and protein production

**DOI:** 10.1038/s41598-018-27687-5

**Published:** 2018-06-22

**Authors:** Fabian M. Arnold, Michael Hohl, Sille Remm, Hendrik Koliwer-Brandl, Sophia Adenau, Sasitorn Chusri, Peter Sander, Hubert Hilbi, Markus A. Seeger

**Affiliations:** 1 Institute of Medical Microbiology, University of Zurich, Zurich, Switzerland; 2 Present Address: Department of Life Sciences, Imperial College London, London, UK; 3 Present Address: Faculty of Traditional Thai Medicine, Prince of Songkla University, Songkla, Thailand

## Abstract

Molecular research on mycobacteria relies on a multitude of tools for the genetic manipulation of these clinically important bacteria. However, a uniform set of vectors allowing for standardized cloning procedures is not available. Here, we developed a versatile series of mycobacterial vectors for gene deletion, complementation and protein production and purification. The vectors are compatible with fragment exchange (FX) cloning, a recently developed high-throughput cloning principle taking advantage of the type IIS restriction enzyme SapI and its capacity to generate sticky trinucleotide ends outside of its recognition sequence. FX cloning allows for the efficient cloning into an entry vector and the facile transfer of the sequenced insert into a variety of destination vectors. We generated a set of mycobacterial expression vectors spanning a wide range of expression strengths, tagging variants and selection markers to rapidly screen for the optimal expression construct in order to purify membrane proteins from the model organism *Mycobacterium smegmatis*. Further, we generated a series of suicide vectors containing two counterselection markers and used them to delete twenty genes encoding for potential drug efflux pumps in *M. smegmatis*. The vectors will further facilitate genetic and biochemical research on various mycobacterial species.

## Introduction

The genus *Mycobacterium* includes pathogens that cause serious diseases in humans, including *Mycobacterium tuberculosis*, the causative agent of tuberculosis. Tuberculosis is one of the world’s most lethal diseases, infecting about one-third of the world’s population and killing around two million people every year^1^. Genetic manipulation of mycobacteria was essential to discover and study cellular processes responsible for virulence and resistance at the molecular level. The last three decades have brought about an impressive number of genetic tools and vectors for the deletion or downregulation of mycobacterial genes^2–9^. However, each of the vectors has its own multiple cloning site, which imposes an obstacle to test different selection markers in parallel. If a gene deletion is conducted, the upstream and downstream homology sequences flanking the target gene (called flanking regions within this manuscript) are usually cloned in two separate steps using different combinations of type I restriction enzymes, which requires adapting the cloning strategy for each gene deletion construct. A notable exception is a recently published procedure based on the restriction enzyme Van91I, which allows cloning of the flanking regions in a single step^9^.

Non-pathogenic and fast-growing *M. smegmatis* has emerged as an important expression host for genes originating from mycobacterial species including *M. tuberculosis*^10–14^. The rationale behind using *M. smegmatis* as expression host is three-fold: (i) mycobacteria have a very high genomic GC content and thus a different codon usage as common expression hosts such as *Escherichia coli*, (ii) the cytoplasm of *M. smegmatis* contains cognate heat shock and other stress-induced proteins, which may improve the proper folding of produced proteins and (iii) the cytoplasmic membrane and in particular the mycobacterial outer membrane has a lipid composition distinct from any other commonly used expression host, which is of high relevance if one aims to produce mycobacterial membrane proteins. The production and purification of mycobacterial membrane proteins is therefore very challenging, which is reflected in a comparatively low number of biochemical and structural studies involving purified mycobacterial transporters, channels and other transmembrane proteins^15–18^. In the field of mycobacteria, three major types of expression vectors have emerged, which exhibit various expression strengths: constitutive expression systems under the strong mycobacterial *hsp60* promoter^19^, and inducible systems based on the tetracycline system^10^, the acetamide promoter^13^, the lac promoter^20^ and a riboswitch-based translational control^21^. The expression strength can be further modulated by using replicating multicopy plasmids containing the mycobacterial origin of replication (*oriM*)^22^ or integrative plasmids which are integrated into the chromosome as a single copy via *attP/attB* recombination^23^. However, a simple cloning scheme, which would allow inserting ORFs fused to different tagging variants for detection (e.g. GFP) and purification (e.g. His-tag) into mycobacterial expression vectors covering a wide range of expression strengths is currently lacking.

With the aim to study mycobacterial membrane transporters at the structural and functional level, we built an efficient cloning, expression and gene deletion platform based on the FX cloning method. The major advantage of FX cloning is that it facilitates fast and efficient cloning of ORFs into desired expression vectors by a robust and simple protocol based on the type IIS restriction enzyme SapI or its isoschizomers. The method proved to be highly efficient in finding optimal expression constructs in *E. coli*, *Lactococcus lactis* and other prokaryotic and eukaryotic expression hosts^24,25^. We constructed mycobacterial FX vectors for i) production and purification of (membrane) proteins using *M. smegmatis* as expression host, ii) fast and robust gene deletion in mycobacterial species, iii) complementation of deletion mutants in the absence of inducer and antibiotics and iv) conditional knockdown based on the pristamycin system. The platform was validated by the expression of an array of mycobacterial membrane transporters in *M. smegmatis* and by the generation of a large number of unmarked gene deletions in *M. smegmatis* and *Mycobacterium marinum*.

## Results

### Overview of the FX-vectors for mycobacterial genetics and protein production

**Figure 1:**
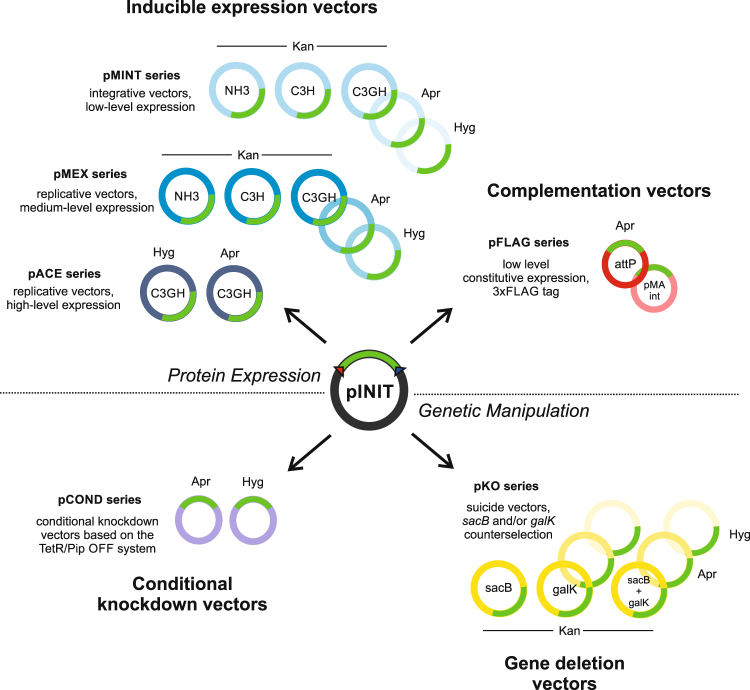
Overview of the mycobacterial FX cloning system. The vectors can be divided into four categories: inducible vectors for protein production and purification, constitutive vectors for gene complementation, vectors for gene deletions and vectors for conditional knockdowns. Open reading frames or flanking homology regions are first cloned into the entry vector pINIT, sequenced, and then transferred by backbone exchange into the mycobacterial destination vectors, which all contain an *E. coli* origin of replication for plasmid propagation.

**Figure 2:**
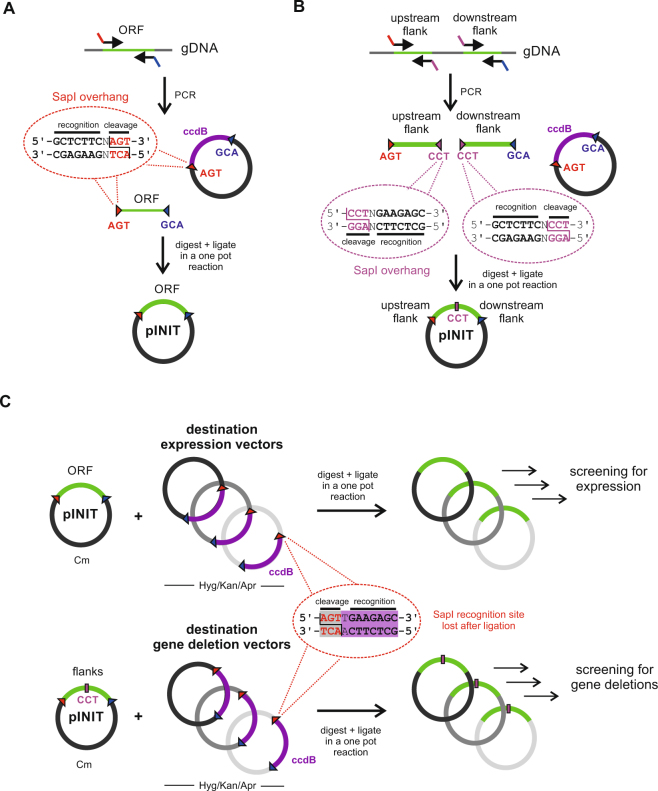
The FX cloning principle. (**A**) Cloning of open reading frames (ORFs). ORFs are PCR amplified from genomic DNA using primers containing SapI overhangs. The purified PCR product is mixed with the pINIT vector containing the *ccdB* killer gene between its SapI restriction sites. Killer gene resistant *E. coli DB3.1* is used to propagate pINIT_ccdB. The mixture is digested with SapI, thereby producing sticky AGT and GCA ends. Upon inactivation of SapI, the fragments are ligated in the same reaction mixture. Only clones containing the ORF as insert survive, while clones harboring back-ligation products containing the *ccdB* killer gene are not viable. (**B**) Cloning of two flanking regions in one step. SapI cleavage is used to introduce the additional sticky end CCT to clone the upstream and downstream flanking regions in a three-fragment ligation reaction into the pINIT backbone. (**C**) Transfer of inserts from pINIT into mycobacterial destination vectors by backbone exchange. The entry vector pINIT harbors a chloramphenicol resistance marker which is absent on the mycobacterial destination vectors. In a single reaction, entry vector pINIT containing an ORF or flanking regions and the destination vector containing the *ccdB* killer gene are digested with SapI, followed by ligation, transformation into *E. coli* and plating using the resistance marker of the destination vector (hygromycin, kanamycin or apramycin). Note that the SapI restriction sites are directed inversely in the destination vectors and therefore disappear after cloning of ORFs or flanking regions.

The vectors in this work can be summarized into four main categories: inducible expression vectors for protein purification, constitutive expression vectors for complementation, conditional knockdown vectors and suicide vectors to generate unmarked gene deletions via two-step recombination (Fig. 1). All vectors are fully compatible with the FX cloning method^24^, permitting the rapid transfer of mycobacterial ORFs or flanking regions from a single entry vector into the respective mycobacterial destination vectors (Fig. 1). In a first step, ORFs are amplified via PCR with appropriate SapI overhangs and cloned into entry vector pINIT in a one-tube reaction including a restriction and a ligation step (Fig. 2A). In order to clone flanking regions into mycobacterial suicide vectors for targeted gene deletions by homologous recombination, two PCR fragments encompassing the upstream and downstream homology sequences are cloned in a three-fragment ligation step into the pINIT backbone as summarized in Fig. 2B. The flanking regions feature an additional SapI cleavage site resulting in a CCT sticky end located at the 3′ and 5′ ends of the upstream and downstream flanking region, respectively, thereby creating an additional unique ligation site. Because of the directionality of the SapI cleavage, the recognition site is lost and only the codon CCT remains as a minimal cloning scar. Upstream flanking regions used for conditional knockdowns follow the same cloning procedure as ORFs for gene expression. Analytical restriction digests are performed to identify positive pINIT clones, which are then confirmed by sequencing. Genetic material of correct pINIT clones can be stored and amplified in this vector, and ORFs as well as homology sequences are subsequently transferred into the desired mycobacterial destination vector by fragment exchange without the need of any further PCR reaction (Fig. 2C). Thereby a major source of introducing unwanted mutations to the cloned sequence is eliminated. A detailed list of all plasmids with information about resistance cassettes and plasmid functionalities is given in Table 1.

**Table 1:** List of mycobacterial destination vectors.

Vector	Description	Purpose	Antibiotic variants	Addgene ID
pMINTC3GH	Integrative vector for expression in mycobacteria based on the *tet* promoter. Contains a C-terminal 3 C cleavage site and a GPF-fusion with a His_10_-tag.	Protein production and purification	Kan Apr Hyg	110076 110078 110077
pMINTC3H	Analogous to pMINTC3GH but with a C-terminal 3 C cleavage site and His_10_ tag. Is devoid of GFP.	Protein production and purification	Kan	110075
pMINTNH3	Analogous to pMINTC3GH but with an N-terminal His_10_ tag and 3 C cleavage site. Is devoid of GFP.	Protein production and purification	Kan	110074
pMEXC3GH	Analogous to pMINTC3GH, but containing *oriM* for episomal replication in mycobacteria.	Protein production and purification	Kan Apr Hyg	110081 110083 110082
pMEXC3H	Analogous to pMINTC3H, but containing *oriM* for episomal replication in mycobacteria.	Protein production and purification	Kan	110080
pMEXNH3	Analogous to pMINTNH3, but containing *oriM* for episomal replication in mycobacteria.	Protein production and purification	Kan	110079
pACEC3GH	Replicative, acetamide-based expression vector containing a C-terminal 3 C cleavage site and a GPF-fusion with a His_10_-tag. Suitable for high-level expression levels amenable for large scale purification of proteins.	Protein production and purification	Hyg Apr	110084 110085
pKO	Suicide vector for generation of gene deletions in mycobacteria. Contains the two genes *sacB* and *galK* for negative selection.	Gene deletion	Kan Apr Hyg	110086 110088 110087
pKO∆galK	Analogous to pKO but without the negative selection gene *galK*.	Gene deletion	Kan Apr Hyg	110089 110091 110090
pKO∆sacB	Analogous to pKO but without the negative selection gene *sacB*.	Gene deletion	Kan	110092
pCOND	Suicide vector based on the TetR/Pip OFF system.	Conditional knockdown	Apr Hyg	110094 110093
pFLAG_attP	Vector for low-level constitutive expression in mycobacteria from the *tet* promoter. Lacks the *tet* repressor. Contains *attP* for chromosomal integration. Lacks the integrase and thus remains stably integrated in the absence of antibiotic selection. Contains a C-terminal 3xFLAG to allow detection of expression levels by Western blotting.	Gene complementation	Apr	110095
pMA_Int	*E. coli* replicative vector which contains the mycophage integrase gene required for the *attP/attB* integration. Is co-transformed *in trans* together with pFLAG_attP, but cannot replicate in mycobacteria, and is thus lost shortly after electroporation.	Gene complementation	Amp	110096
pFLAG_OriM	Analogous to pFLAG_attP, but containing *oriM* instead of *attP*, thus being a replicative vector.	Gene complementation	Hyg	110097

### Protein production using the inducible vectors pMINT, pMEX and pACE

**Figure 3:**
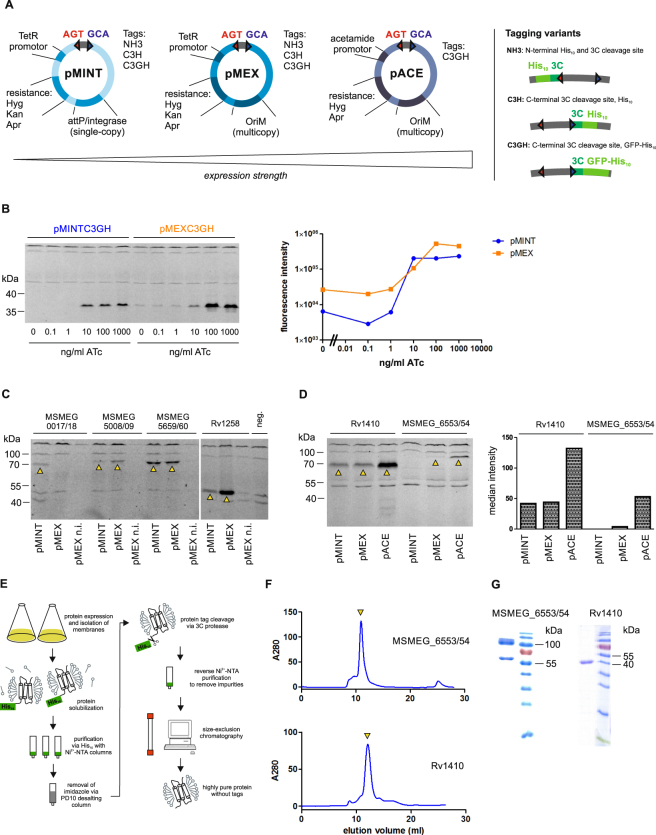
Mycobacterial destination vector series for protein production and purification. (**A**) Schematic overview of the inducible expression vectors, ordered by expression strength. Relevant features are schematically depicted for each vector type. The three tagging variants are shown schematically. (**B**) Expression of a designed ankyrin repeat protein (DARPin) in integrative pMINTC3GH and replicative pMEXC3GH at varying ATc concentrations. The left panel shows expression levels detected by *in gel* GFP fluorescence of cell lysates separated by SDS-PAGE. The band between 35 and 40 kDa corresponds to the DARPin-GFP fusion protein. The right panel shows the quantification of DARPin-GFP fluorescence intensities. (**C**) Expression tests of the heterodimeric ABC transporters MSMEG_0017/18, MSMEG_5008/09, MSMEG_5659/60 and the MFS transporter Rv1258 cloned as GFP fusion proteins in expression vectors pMINTC3GH and pMEXC3GH. Total cell lysates were separated by SDS-PAGE and protein production was visualized by *in gel* fluorescence. Detected transporter-GFP fusion proteins are marked with arrows. Leaky expression was assessed using the pMEXC3GH constructs in the absence of inducer (n.i. stands for “not induced”). Supernatant of wildtype *M. smegmatis* was loaded as a negative control to assess background fluorescence (“neg”). (**D**) Side-by-side comparison of production levels of the MFS transporter Rv1410 and the heterodimeric ABC transporter MSMEG_6553/54 expressed in pMINTC3GH, pMEXC3GH and pACEC3GH. The left panel shows the *in gel* fluorescence of total cell lysates separated by SDS-PAGE. Bands corresponding to the expected size of the transporter-GFP fusion protein are marked with an arrow. The right panel shows the quantification of protein production. (**E**) Schematic overview of preparative purification of tag-less Rv1410 and MSMEG_6553/54 expressed in pACEC3GH for structural biology purposes. (**F**) Size exclusion chromatography (SEC) profiles of purified Rv1410 and MSMEG_6553/54. Elution peaks corresponding to Rv1410 and MSMEG_6553/54 are marked by a yellow arrow. (**G**) SDS-PAGE analysis and Coomassie staining of transporters as eluting from SEC shown in (**F**). Of note, the two bands observed for MSMEG_6553/54 correspond to MSMEG_6553 (upper band at around 100 kDa) and MSMEG_6554 (lower band at around 65 kDa).

To be able to screen for the most suitable production level of a protein (and in particular a membrane protein), our aim was to construct vectors with low, medium and high gene expression levels. Additionally, we aimed for different tagging variants and resistance markers. To achieve this goal, we cloned the vector series pMINT, pMEX and pACE, which all can be replicated in *E. coli* for cloning purposes and plasmid amplification. The pMINT series is based on the tetracycline expression system^10^ and contains the phage L5 *attP* site and integrase for site-specific recombination with the *attB* site on the mycobacterial chromosome^23^, which allow for a controlled, low-level expression of proteins. The pMEX series is also based on the same tetracycline expression system, but comprises multicopy vectors harboring a mycobacterial origin of replication (*oriM*), thereby conferring higher expression levels than the pMINT vectors^10^. The episomal pACE vectors are based on the stronger acetamide promotor, allowing for the highest production level of proteins^13^. An overview of these vectors with their different tags and resistance markers is given in Fig. 3A. The tetracycline system, on which pMINT and pMEX are based, was described to be very tight^10^. To test pMINT and pMEX in *M. smegmatis* and assess the production level difference between pMINT and pMEX we chose a designed ankyrin repeat protein (DARPin), which is a stable soluble protein produced at high levels in *E. coli*^26^. The DARPin gene was cloned first into the pINIT vector and then transferred by FX cloning into pMINTC3GH and pMEXC3GH, thereby attaching a 3 C protease cleavage site, GFP and a His_10_-tag to the C-terminus. The DARPin constructs were then expressed overnight in *M. smegmatis* at varying concentrations of anhydrotetracycline (ATc). The quantification of production levels via *in gel* fluorescence of the GFP-fusion protein shows that the expression can be titrated with both vectors over a broad range of ATc concentrations (Fig. 3B). The replicative pMEX construct allows for higher production at cost of a higher basal production level. To test production of challenging proteins, four mycobacterial ORFs encoding for membrane transporters were cloned via entry vector pINIT into the destination vectors pMINTC3GH and pMEXC3GH, again attaching (cytoplasmatically located) GFP to the C-termini of these ORFs. The transporters were produced overnight in *M. smegmatis* by adding 100 ng/ml ATc and protein production was assessed again by *in gel* fluorescence of the GFP-fusion proteins (Fig. 3C). All transporters were produced as indicated by the fluorescent band at the expected height corresponding to the respective transporter-GFP fusion. Of note, GFP fluorescence indicates proper folding of the membrane proteins, because it only folds correctly if the fused membrane protein is correctly folded as well^27^. With the exception of MSMEG_0017/18, pMEX constructs produced to a higher level than the respective pMINT constructs. The non-induced pMEX control cultures did not exhibit any detectable recombinant protein production, indicating that these vectors are sufficiently tight for the production of membrane proteins without risking toxicity problems. Finally, we tested production of two transporters with the pACE vector. We chose the major facilitator superfamily (MFS) transporter Rv1410, as well as the heterodimeric ABC exporter MSMEG_6553/54. We performed an overnight expression with 0.4% acetamide for induction, and analyzed protein production by *in gel* fluorescence of the transporter-GFP fusion. In parallel, we performed the expression with the respective pMINTC3GH and pMEXC3GH constructs induced with 200 ng/ml ATc. The *in gel* fluorescence analysis showed, that for both transporter proteins markedly higher production levels were achieved with the pACEC3GH construct (Fig. 3D). To investigate whether the production levels using the pACE constructs were high enough for preparative-scale purification, the GFP-tagged membrane proteins Rv1410 and MSMEG_6553/54 were produced in 9 and 4.5 liter of 7H9 medium, respectively. The cells were lysed, the membrane fraction was harvested by ultracentrifugation, and the membrane transporters were extracted using the detergent n-dodecyl-β-D-maltopyranoside (β-DDM) and purified using Ni^2+^-NTA chromatography via the C-terminal His_10_-tag. Subsequently, GFP-His_10_ was cleaved off using 3 C protease and removed in a reverse Ni^2+^-NTA chromatography step (a schematic overview of the purification is given in Fig. 3E). Finally, untagged protein was polished by size exclusion chromatography (SEC) and eluted as single, monodisperse peak at a retention volume corresponding to the monomer in case of Rv1410 or a heterodimer in case of MSMEG_6553/54 (Fig. 3F). The protein yields were 1 mg/batch and 0.8 mg/batch for Rv1410 and MSMEG_6553/54, respectively, and the transporters were highly pure as judged from SDS-PAGE analysis (Fig. 3G).

### Complementation vector pFLAG

**Figure 4:**
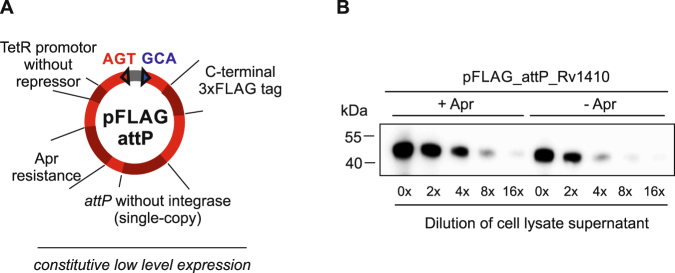
Mycobacterial complementation vector pFLAG for constitutive low-level expression. (**A**) Schematic overview of the pFLAG vector depicting relevant features. (**B**) Detection of Rv1410-3xFLAG produced from integrative vector pFLAG_attP in the presence or absence of apramycin. Total cell lysate supernatants were separated as a two-fold dilution series by SDS-PAGE and Rv1410-3xFLAG was detected by Western blotting using an anti-FLAG antibody. The uncropped Western blot image is shown in Supplementary Fig. S1.

In functional studies, gene deletion mutants usually need to be complemented with the wildtype gene *in trans* to demonstrate that the genetic manipulation exclusively affected the targeted gene as indicated by a reversion of the phenotype. An ideal complementation vector (i) exhibits a moderate promoter strength to avoid toxicity effects from overexpression, (ii) contains a constitutive promoter and thus functions in the absence of inducers, (iii) is stably integrated as a single copy on the genome even in the absence of selective pressure and (iv) fuses a short tag to the complemented ORF to enable highly sensitive detection of protein production. To meet these requirements, we generated pFLAG_attP. It harbors the *tet*-promoter as pMINT, but lacks the *tet*  repressor gene, rendering the promoter constitutively active at a moderate level. The plasmid further contains an *attP* site for chromosomal integration, but lacks the integrase gene, which allows for stable integration of pFLAG_attP into the mycobacterial genome if the integrase gene is provided on a separate suicide vector *in trans*^28^. Finally, pFLAG_attP fuses a triple FLAG-tag (3xFLAG) to the C-terminus of cloned ORFs (Fig. 4A), enabling the highly sensitive detection of protein production even from minute amounts of bacterial cells by Western blotting. To demonstrate the utility of the 3xFLAG-tag, we transferred the gene encoding *M. tuberculosis* Rv1410 from the pINIT vector into pFLAG_attP using FX cloning. The resulting vector was propagated in *E. coli* and then electroporated into *M. smegmatis* by co-electroporation *in trans* with the L5 bacteriophage integrase present on the suicide vector pMA_Int, which can neither replicate nor integrate in mycobacteria. Stable integrants were obtained by selecting on apramycin plates and confirmed by colony PCR. One integrant was grown to stationary phase in the presence or absence of apramycin, and a two-fold dilution series of total protein from a small amount of cells was separated by SDS-PAGE and analyzed by Western blotting for production using an anti-FLAG antibody (Fig. 4B). Production of Rv1410 was clearly detected regardless of whether the culture was grown with or without apramycin, supporting the notion that the pFLAG vectors are kept stably integrated in the genome even without antibiotic selection. However, protein production was slightly lower in the absence of apramycin.

### Generation of deletion mutants with the pKO vectors

**Figure 5:**
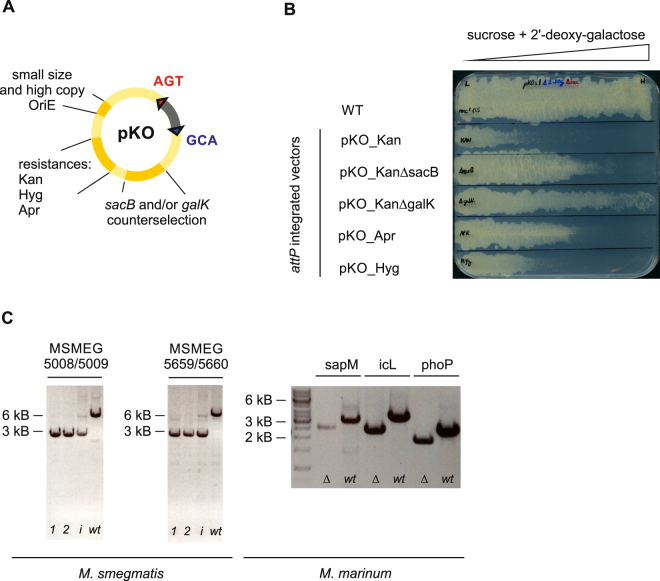
Mycobacterial destination vector series pKO for the generation of unmarked gene deletions. (**A**) Schematic overview of pKO vectors depicting relevant features. (**B**) Counterselection test of pKO vectors. *M. smegmatis* harboring different pKO versions stably integrated via *attP/attB* recombination was streaked on gradient plates containing an increasing amount (from left to right) of sugars for counterselection. Wildtype *M. smegmatis* was streaked as a control. (**C**) Generation of unmarked gene deletions of the heterodimeric ABC transporters *msmeg_5008/09* and *msmeg_5659/60* in *M. smegmatis* as well as *sapM*, *icL* and *phoP* in *M. marinum*. Shown are agarose gels of PCR products amplified from genomic DNA. For *M*. *smegmatis* (left), PCR products amplified from two gene deletion clones (1 and 2, expected size of ~3 kB), the strain containing the integrated suicide plasmid as obtained after the first recombination step (*i*, expected sizes of ~3 kB and ~6 kB), and wildtype controls (*wt*, expected size of ~6 kB) are shown. In strains containing the integrated suicide plasmid, the 3 kB fragment is amplified more efficiently than the 6 kB fragment. For *M. marinum* (right), gene deletions and wildtype controls are shown. The expected sizes of the PCR products for mutant/wildtype are as follows: For *sapM* ~2.5 kB/ ~3 kB, for *icL* ~2.4 kB/ ~3.2 kB and for *phoP* ~1.8 kB/ ~2.5 kB.

The process of generating unmarked gene deletions with suicide plasmids is well established, but has some drawbacks: first, the genetic loci upstream and downstream of the target gene must be combined into one suicide vector over several cloning steps; second, the first recombination step is inefficient and requires large amounts of suicide vector for electroporation to obtain a few clones; and third, the second recombination step often yields false positive clones due to mutations in the counterselection gene. To address these issues we cloned the pKO vectors series (Fig. 5A), which offers the following three advantages: first, the flanking regions are cloned in one single cloning step into entry vector pINIT and are then transferred to any of the pKO destination vectors as described in Fig. 1C, which allows to test the three commonly used antibiotic selection markers conferring resistance towards hygromycin, kanamycin and apramycin with minimal effort; second, pKO vectors are relatively small (between 5.7 to 6.2 kb) and have a high copy number in *E. coli*, facilitating the production of large amounts of these vectors for electroporation; third, pKO vectors harbor the two counterselection genes *sacB* and *galK*, allowing for a tight counterselection^29^. To test the counterselection strength of the pKO vectors, we introduced the *attP* site instead of flanking regions. The pKO_*attP* vectors were then stably introduced into *M. smegmatis* by co-electroporation *in trans* with the L5 bacteriophage integrase present on the suicide vector pMA_Int. Stable integrants were first selected on agar plates containing the appropriate antibiotic (hygromycin, kanamycin or apramycin) and confirmed by colony PCR. As a control, either the *galK* or the *sacB* gene was deleted on the pKO vector carrying the kanamycin marker (pKO_KanΔgalK, pKO_KanΔsacB) and was integrated into the genome of *M. smegmatis*, accordingly. Subsequently, the integrants were grown to stationary phase, and streaked onto plates containing a gradient of the counterselection sugars sucrose and 2-deoxy-galactose. As expected, counterselection using both markers resulted in a more efficient killing of *M. smegmatis* than integrants harboring only *sacB* or *galK* (Fig. 5B). In parallel tests using the same flanking regions in pKO vectors carrying the hygromycin, kanamycin or apramycin resistance markers, respectively, we found that pKO_Apr had the best performance in *M. smegmatis*. For *M. marinum*, only pKO_Hyg was tested. In *M. smegmatis*, hygromycin performed rather poorly, because there was substantial background growth on 7H10 plates, and kanamycin led to fewer positive clones compared to apramycin. However, it is expected that this set of antibiotic markers likely performs differently in other mycobacterial species, and therefore, we advise to test the entire pKO series to identify the optimal selection marker. After rigorously assessing positive selection and counterselection, we used the pKO_Apr vectors to generate 20 unmarked gene deletions in *M. smegmatis* encoding potential multidrug efflux pumps. The transporters belong to four protein superfamilies, the major facilitator superfamily (MFS), the small multidrug resistance (SMR), the multidrug and toxin extrusion (MATE) and the ATP binding cassette (ABC) transporters (Table 2). Gene deletions were verified by PCR amplifying the deleted region (Fig. 5C). These unmarked gene deletion mutants were tested for increased antibiotic susceptibility against twelve antibiotics and the drug efflux dye ethidium. This extensive analysis revealed an 8-fold increased ethidium susceptibility for cells containing a deletion in *msmeg_6225* (encoding the previously described MFS drug transporter LfrA^30^) and a 2-fold increased susceptibility for cells devoid of *msmeg_3705*, encoding an MFS transporter previously described as drug efflux pump^31^. Remarkably, our analysis did not reveal increased antibiotic susceptibilities for any other gene deletion. This finding was surprising, because some bacteria contain dominant drug efflux pumps such as the AcrAB-TolC system of *E. coli* or the heterodimeric ABC exporter LmrCD of *L. lactis*, whose chromosomal deletion leads to a pronounced increase of drug susceptibility^32,33^. Our finding indicates that *M. smegmatis* either contains a multitude of redundant drug efflux pumps, which compensate for each other in case one is deleted, or drug efflux does not play a dominant role for antibiotic sensitivity in this organism. To test the applicability of the pKO vector series for other mycobacterial species, the three genes *sapM* (*mmar_1216*), *icL* (*mmar_0792*) and *phoP* (*mmar_4942*) were deleted in *M. marinum*. To our knowledge, so far only two publications describe the successful generation of defined gene deletions mutants in *M. marinum*^34,35^, but both are based on phage transduction and not suicide vectors, and thus require resolvase genes to render the deletions unmarked^36^. We used the vector pKO_Hyg to generate unmarked gene deletions of these genes, and gene deletions were confirmed by PCR and Sanger sequencing (Fig. 5C).

**Table 2:** MIC values (µg/ml) of multidrug transporter gene deletion mutants compared to wildtype *M. smegmatis*.

	ABC								MATE	MFS							SMR				wild type
	ΔMSMEG_0017/0018	ΔMSMEG_1502/1504	ΔMSMEG_1642	ΔMSMEG_4380	ΔMSMEG_5008/5009	ΔMSMEG_5075/5076	ΔMSMEG_5659/5660	ΔMSMEG_6509/6510	ΔMSMEG_2631	ΔMSMEG_3563	ΔMSMEG_3705	ΔMSMEG_5046	ΔMSMEG_5187	ΔMSMEG_5670	ΔMSMEG_6225 (LfrA)	ΔMSMEG_6390	ΔMSMEG_3670	ΔMSMEG_3672	ΔMSMEG_3815	ΔMSMEG_6221	
Isoniazid	200	200	200	200	200	200	200	200	200	200	200	200	200	200	200	200	200	200	200	200	200
Rifampicin	8	8	8	8	8	8	8	8	8	8	8	8	8	8	8	8	8	8	8	8	8
Ethambutol	>10	>10	>10	>10	>10	>10	>10	>10	>10	>10	>10	>10	>10	>10	>10	>10	>10	>10	>10	>10	>10
Streptomycin	0.25	0.25	0.25	0.25	0.25	0.25	0.25	0.25	0.25	0.25	0.25	0.25	0.25	0.25	0.25	0.25	0.25	0.25	0.25	0.25	0.25
Kanamycin	2.5	2.5	2.5	2.5	2.5	2.5	2.5	2.5	2.5	2.5	2.5	2.5	2.5	2.5	2.5	2.5	2.5	2.5	2.5	2.5	2.5
Ofloxacin	0.6	0.6	0.6	0.6	0.6	0.6	0.6	0.6	0.6	0.6	0.6	0.6	0.6	0.6	0.6	0.6	0.6	0.6	0.6	0.6	0.6
Ciprofloxacin	0.6	0.6	0.6	0.6	0.6	0.6	0.6	0.6	0.6	0.6	0.6	0.6	0.6	0.6	0.6	0.6	0.6	0.6	0.6	0.6	0.6
Ampicillin	125	125	125	125	125	125	125	125	125	125	125	125	125	125	125	125	125	125	125	125	125
Chloramphenicol	32	32	32	32	32	32	32	32	32	32	32	32	32	32	32	32	32	32	32	32	32
Tetracycline	1.2	1.2	1.2	1.2	1.2	1.2	1.2	1.2	1.2	1.2	1.2	1.2	1.2	1.2	1.2	1.2	1.2	1.2	1.2	1.2	1.2
Vancomycin	2.5	2.5	2.5	2.5	2.5	2.5	2.5	2.5	2.5	2.5	2.5	2.5	2.5	2.5	2.5	2.5	2.5	2.5	2.5	2.5	2.5
Novobiocin	16	16	16	16	16	16	16	16	16	16	16	16	16	16	16	16	16	16	16	16	16
Ethidium	6	6	6	6	6	6	6	6	6	6	**3**	6	6	6	**0.75**	6	6	6	6	6	6

The transporters are sorted according to transporter families. MIC determinations were carried out as technical duplicates. In the rare event that the two values differed, the higher of the two MIC values was reported.

### Generation of conditional knockdowns

**Figure 6:**
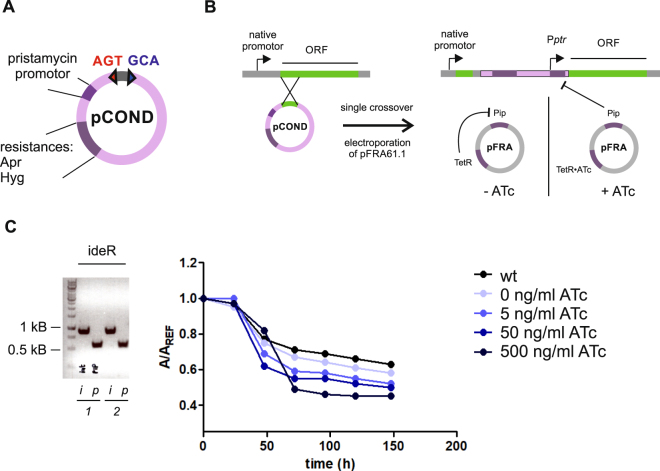
Mycobacterial destination vectors pCOND for generation of conditional knockdowns. (**A**) Schematic overview of pCOND vectors. (**B**) The native promoter of an ORF is first replaced by P_*ptr*_ using homologous recombination. The Pip protein repressing P_*ptr*_ is encoded on the pFRA61.1 vector under the control of the inducible tetracycline promoter system. (**C**) Left, PCR to test integration (*i*) of the pCOND vector into *M. smegmatis* for two clones of *ideR* conditional knockdowns, and PCR to test for the presence of the *pip* promotor (*p*). Right, CAS assay to monitor the biosynthesis and secretion of the siderophores for the *ideR* conditional knockdown strain grown at different concentrations of ATc. Higher ATc concentrations lead to increased siderophore production, resulting in lower A/A_ref_ values in the CAS assay.

If an essential gene is targeted, deletion mutants cannot be obtained. Therefore, conditional knockdown mutants are generated to study the function of essential genes. Knockdowns can be generated either at the transcriptional level by using for example the tetracycline repressor (TetR)-controlled expression system^11^ or at the protein level by tagging an essential protein for proteolytic degradation^6^. Here, we opted for the TetR/Pip OFF repressible promoter system, because of its strong and tight gene activation and repression^5^. In this system, the essential gene is placed under the control of the pristamycin promoter (P_*ptr*_) which is tightly repressed by the pristamycin repressor (Pip). Expression of the *pip* gene in turn is under the control of the tetracycline repressor TetR, which is constitutively produced. In the absence of ATc, production of Pip is repressed and the essential gene under the control of P_*ptr*_ is expressed. In the presence of ATc, Pip is produced, represses P_*ptr*_ and leads to a depletion of the essential gene product^5^ (Fig. 6). We adapted this system to our FX cloning platform and constructed pCOND. This suicide vector carries P_*ptr*_ followed by SapI sites to clone the first 500–600 bp of an essential gene of interest under the control of P_*ptr*_. We constructed pCOND containing either a hygromycin or an apramycin resistance marker with an *E. coli* origin of replication for cloning and plasmid propagation (Fig. 6). For a project on siderophore transporters, we aimed to engineer an *M. smegmatis* strain, which produces high amounts of the soluble siderophore carboxymycobactin^37^. A strain containing a gene knockout of the iron regulator *ideR* was reported to exhibit de-repressed siderophore biosynthesis^38^. However, our own attempts to delete *ideR* were not successful. Therefore, we used pCOND to generate a conditional knockdown of *ideR* in *M. smegmatis*. To this end, the first 513 bp of the *ideR* gene were cloned by FX cloning via pINIT into pCOND_Apr. The construct was then integrated into the genome of *M. smegmatis* by homologous recombination, thereby replacing the native promoter of *ideR* by P_*ptr*_. Subsequently, the plasmid pFRA61.1, which carries TetR and the gene encoding the Pip repressor under a promoter repressed by TetR as described previously^5^, was integrated via *attP/attB* recombination into this *M. smegmatis* strain. The resulting conditional knockdown strain was grown in minimal medium in the presence of increasing ATc concentrations, and siderophore secretion into the medium was measured with the chrome azurol S (CAS) assay^39^. In this assay, empty siderophore captures Fe^3+^ from a colored dye-iron complex, leading to an absorption decrease as a measure of siderophore production. As expected, the conditional *ideR* strain showed increased siderophore production upon induction with increasing ATc concentrations compared to the wildtype strain (Fig. 6C).

## Discussion

Mechanistic research on mycobacteria critically depends on genetic tools to generate deletion mutants and to express wildtype and mutated genes under the control of inducible promoters. In the past three decades, a plethora of vectors to genetically manipulate mycobacteria has therefore been created and successfully used. In particular for biochemical and structural studies on membrane proteins, efficient tools for protein production in and purification from the mycobacterial workhorse species *M. smegmatis* are highly relevant. However, standardized cloning and expression systems for efficient construct screening as they are available for other commonly used expression hosts such as *E. coli*^27^ and *Saccharomyces cerevisiae*^40^ did not exist for *M. smegmatis*. In this work, we adapted a broad range of mycobacterial vectors to be used with the efficient and cost-effective FX cloning system^41^. The protocol starts with the cloning of ORFs or homology regions into an entry vector following the same robust cloning protocol using just one single type IIS restriction enzyme, SapI (or its isoschizomers BspQI and LguI). Due to the high GC content, mycobacterial genes are notoriously difficult to amplify by PCR. Once the genes or flanking regions of interest are safely introduced in the entry vector and their sequence confirmed, they can be transferred by fragment exchange into any of the mycobacterial FX vectors and do not need to be amplified again by PCR to introduce new restriction sites compatible with the destination vector. Thereby, FX cloning removes a major source of errors and avoids tedious troubleshooting.

At the heart of our platform are three vector series for (membrane) protein overproduction in *M. smegmatis*, ranging from low level gene expression (pMINT vectors), medium level expression (pMEX vectors) to high level expression (pACE) amenable for preparative scale protein purification. Various tagging variants exist for this vector series, introducing His_10_-tags for Ni^2+^-NTA purification either N- or C-terminally fused to the cloned ORF, which can be cleaved by the highly efficient 3 C protease to obtain untagged protein for structural biology purposes. As an option, the ORF can be expressed fused to GFP for high-throughput expression screening by *in gel* fluorescence^27^. Last but not least, the expression vector series exists for three commonly used selection markers conferring hygromycin, kanamycin and apramycin resistance, respectively. For *M. smegmatis*, apramycin emerged as our preferred antibiotic, because it is inexpensive, highly selective and does not affect bacterial growth rate to the extent as kanamycin.

The pFLAG vector was found to be ideally suited for gene deletion complementation in a variety of projects involving transporter knockouts (unpublished results). In pFLAG, the cloned gene is constitutively expressed from the medium strength tetracycline promoter, and consequently, no inducer is required during a complementation experiment. Although constitutive expression does not allow for tuning expression levels by optimizing the inducer concentration, we so far did not experience problems in our own complementation attempts. Of note, vectors of the pMINT series are an attractive alternative for complementation studies, because they are integrative vectors akin to pFLAG, but gene expression is under the control of the inducible TetR repressor^10^. Importantly, pFLAG_attP lacks the integrase gene, which permits its stable integration into the genome by transiently providing the integrase on a suicide vector *in trans*^28^. This allows for complementation experiments in the absence of antibiotics, thereby alleviating side effects attributed to cellular stress induced by antibiotics. Although expression levels obtained with pFLAG are rather low, protein production could be readily detected using the highly sensitive anti-FLAG antibody, which recognizes the C-terminally fused 3xFLAG-tag.

The pKO vector series allows for efficient generation of unmarked gene deletions. The FX cloning method was adapted to clone the flanking homology regions in a single cloning step using SapI as the only restriction enzyme. A similar approach to clone flanking regions was recently chosen using the type IIP restriction enzyme Van91I^9^. The size of the pKO vectors was kept minimal, and they replicate as high copy vectors in *E*. *coli*, facilitating the preparation of highly concentrated plasmid stocks for efficient electroporation into mycobacteria. The two marker genes *sacB* and *galK* ensure tight counterselection and minimize the number of false positive clones in the second recombination step. The pKO vectors come with the three most commonly used selection markers conferring hygromycin, kanamycin and apramycin resistance. Thus, the most appropriate antibiotic can be chosen depending on the mycobacterial species.

Finally, we also generated the pCOND series containing apramycin or hygromycin resistance markers, which was successfully used to generate a conditional knockdown of the iron regulator protein IdeR in *M. smegmatis* to increase the production and secretion of the siderophore carboxymycobactin^37^.

We successfully validated these mycobacterial FX vectors in our own and other selected labs for membrane protein production and purification. Further, we generated a large set of unmarked gene deletions and carried out complementation studies. To our knowledge, this is the first reported uniform and consistent cloning platform spanning protein production and knockout generation in mycobacteria. This resource is available on Addgene (www.addgene.org) for the growing community of biochemists and structural biologists aiming at the molecular investigation of mycobacterial (membrane) proteins. We anticipate that this vector series will facilitate research in other labs as it permits to screen multiple expression and gene deletion constructs in parallel, all based on the cost-effective open-access FX cloning system with its straightforward primer design and high cloning efficiencies.

## Materials and Methods

### Strains, media and antibiotics concentrations

The *E. coli* XL1-Blue strain was used for cloning the mycobacterial FX vectors. Luria Broth (LB) was used for liquid cultures, and Luria Broth Agar (LB Agar) for plates. Antibiotics were used in the following concentrations for *E. coli*: ampicillin (Amp) 100 µg/ml, kanamycin (Kan) 50 µg/ml, chloramphenicol (Cm) 25 µg/ml, apramycin (Apr) 50 µg/ml and hygromycin (Hyg) 100 µg/ml. *M. smegmatis* mc^2^ 155 was used in all experiments involving *M. smegmatis*. 7H9 with OADC supplement was used for liquid cultures, and 7H10 with OADC supplement was used for plates. Antibiotics were used in the following concentrations for *M. smegmatis*: kanamycin 25 µg/ml, apramycin 25 µg/ml and hygromycin 50 µg/ml. If not stated otherwise, cells were incubated and grown at 37 °C. *M. marinum M* (ATCC BAA-535) was used for gene deletion generation in *M. marinum* and grown at 30 °C.

### Construction of the tetracycline-inducible vectors pMINT and pMEX

The backbone of the TetR-based expression vector pGMCKq1-10M1-sspBmyc^6^ (Addgene) was amplified with the 5′-phosphorylated primers pGMCK Fw and pGMCK Rv, yielding a linear PCR fragment (for primer sequences see Table S1). Another linear fragment was generated by PCR using the FX vector pBXC3GH^24^ as a template with the primers pBXC3GH Fw and pBXC3GH Rv. Both linear PCR products were ligated by blunt ligation. After transformation, clones carrying the desired ligation construct resulting in the vector pMINTC3GH were selected based on restriction digest analysis. The vector pMINTC3H was generated by amplifying pMINTC3GH with the primers pMINTC3H Fw and pMINTC3H Rv. The linear PCR fragment was self-ligated by blunt-end ligation, yielding the vector pMINTC3H. The vector pMINTNH3 was constructed analogously to the vector pMINTC3GH, by blunt-end ligation of the backbone of pGMCKq1-10M1-sspBmyc with the linear fragment amplified from the FX vector pBXNH3 using the primers pBXNH3 Fw and pBXNH3 Rv. To generate the respective pMEX vectors (pMEXC3GH, pMEXC3H and pMEXNH3) the *oriM* was amplified from the vector pMYC by PCR using the primers pMYC_OriM Fw and pMYC_OriM Rv. Vector pMYC (Addgene plasmid #42192) is a pSD26 derivative^12^. The three pMINT vectors were amplified by using the primers pMINT_OriM Fw and pMINT_OriM Rv. PCR fragments of the three vector backbones and *oriM* were digested by BbsI and XbaI and ligated accordingly to form the vectors pMEXC3GH, pMEXC3H and pMEXNH3. Being based on backbone of vector pGMCKq1-10M1-sspBmyc, the initial set of pMINT and pMEX constructs harbors a kanamycin resistance cassette. To produce the respective pMINT and pMEX vectors harboring an apramycin or a hygromycin resistance gene, the pMINT and pMEX backbones were amplified using the 5′-phosphorylated primers pMINT/MEX_bb Fw and pMINT/MEX_bb Rv. The genes encoding the apramycin and hygromycin resistance were amplified from the vectors pSET152^42^ and pMYC, respectively, using the primers pSET_apr Fw and pSET_apr Rv, and pMYC_hyg Fw and pMYC_hyg Rv. The linear PCR fragment forming the pMINT and pMEX backbone was then obtained by blunt-end ligation to the respective PCR fragments containing the apramycin and hygromycin resistance genes to yield the pMINT and pMEX vectors with apramycin and hygromycin resistance markers.

### Construction of the acetamidase based expression vector pACE

The backbone of the acetamide-inducible expression vector pMYC was amplified using the 5′-phosphorylated primers pMYC_bb Fw and pMYC_bb Rv. The same PCR fragment amplified from the FX vector pBXC3GH mentioned above was ligated via blunt ends to the amplified pMYC backbone. After transformation clones carrying the desired ligation construct resulting in pACE_Hyg were selected by restriction digest analysis. To make the pACEC3GH vector fully compatible with the FX protocol, the internal SapI restriction site located on the *amiS* gene of the acetamidase operon was altered using the QuikChange primers pACE_sapI Fw and pACE_sapI Rv. To generate the vector pACE_Apr, pACE_Hyg was amplified with the phosphorylated primers pACE_Apr Fw and pACE_Apr Rv and ligated via blunt ends with the PCR product amplified with the primers pSET_apr Fw and pSET_apr Rv from pSET152.

### Construction of complementation vector pFLAG

Three different vectors were used to construct the pFLAG_attP vector: pMINT∆TetR, pBXCF3GH and pCOND with apramycin resistance (see below). The vector pMINT∆TetR was obtained by removing the Tet repressor (TetR) of pMINTC3GH using the 5′-phosphorylated primers pMINT∆TetR Fw and pMINT∆TetR Rv, followed by self-ligation of the resulting PCR product. The vector pBXCF3GH was generated by amplifying the pBXC3GH vector with the 5′-phosphorylated primer pair pBXCF3GH Fw and pBXCF3GH Rv. The linear PCR product was self-ligated to obtain pBXCF3GH, which is a pBXC3GH vector containing a 3xFLAG tag sequence upstream of the 3 C cleavage site. To construct pFLAG_attP, three linear PCR fragments were amplified: the first fragment from pMINT∆TetR with primers pMINT∆TetR_bb Fw and pMINT∆TetR_bb Rv, the second fragment from pBXCF3GH with the primers pBXCF3GH_bb Fw and pBXCF3GH_bb Rv, and the third fragment from pCOND_Apr (see below) with the primers pCOND_APR_bb Fw and pCOND_APR_bb Rv. The first two fragments were used as megaprimers for an overlap extension PCR using primers pMINT∆TetR_bb Fw and pBXC3F3GH_bb Rv. The resulting product was cloned by blunt-end ligation with the third fragment to generate the vector pFLAG_attP. Correct clones were identified by restriction analysis. pFLAG_attP lacks the phage integrase gene for specific recombination via the *attP/attB* site. Therefore, we constructed the vector pMA_SapI, which is a pMA vector (Thermo Fisher Scientific) adapted to FX cloning via two SapI sites. To this end, we amplified pMA with the 5′-phosphorylated primers pMA_FX Fw and pMA_FX Rv and blunt-end ligated the PCR product to generate the vector pMA_SapI. Subsequently, we amplified the integrase gene from pMINTC3GH with the primers Int Fw and Int Rv and cloned it into pMA_SapI to generate the vector pMA_Int, which was used for all subsequent electroporations where the integrase needs to be delivered *in trans*.

### Construction of the pKO vector series

The pKO vector containing the kanamycin resistance marker was constructed in two steps. First, two linear PCR fragments were amplified from the vectors pMA_SapI and pDB77^29^ using 5′-phosphorylated primer pairs pMA_SapI Fw and pMA_SapI Rv, and pDB77 Fw and pDB77 Rv, respectively, followed by the blunt-end ligation of the resulting PCR products. The internal SapI site on pMA_DB77 was removed using the QuikChange primers pMA_DB77 Fw and Rv, yielding the new vector pKO∆sacB. In a second step, the backbone of vector pKO∆sacB was amplified with the primer pair pKO∆sacB Fw/pKO∆sacB Rv. The *sacB* gene was amplified from the FX vector pINIT^24^ with the primers pINIT_sacB Fw and pINIT_sacB Rv. Both fragments were digested with BbsI and XbaI and then ligated to form the vector pKO_Kan, harboring the counterselection genes *galK* and *sacB* along with a kanamycin resistance gene. Additionally, pKO vectors with hygromycin and apramycin resistance (pKO_Hyg and pKO_Apr) were generated by cloning the hygromycin and apramycin genes (as used to construct the pMINT series) via XbaI and NcoI restriction sites. Finally, the pKO∆galK vectors containing either kanamycin, hygromycin or apramycin resistance markers were generated by BamHI digest to excise the *galK* gene followed by self-ligation.

### Construction of the pCOND vectors

The vector pTE (Addgene plasmid #20320) was amplified with the 5′-phosphoraylated primers pTE_bb Fw and pTE_bb Rv, and the PCR product was blunt-end ligated to the same PCR product containing the apramycin cassette described in the construction of the pMINT and pMEX vectors, resulting in the vector pTE_Apr. Both pTE and pTE_Apr were used as a backbone for the construction of the pCOND vector. The backbone of the pTE vectors was amplified using the forward primer pTE Fw and pTE Rv, both containing a BamHI overhang. The pristamycin promoter (P_*ptr*_) was amplified by PCR from vector pFRA50^5^ using the primer pair pFRA50 Fw and pFRA50 Rv, both containing a BamHI overhang. Both PCR products were then digested with BamHI and ligated to form pCOND (with apramycin and hygromycin resistance, depending on the pTE backbone).

### Cloning of ORFs for protein production

ORFs encoding genes of interest were PCR amplified using Q5 DNA polymerase (NEB) from genomic DNA for *M. smegmatis* and *M. marinum*, and from the H37Rv bacmid library^43^ for *M. tuberculosis*. Amplification was performed with FX-compatible primers designed with the FX-primer tool (www.fxcloning.org). The amplicons were cloned into the pINIT vector according to the FX protocol and sequenced. In brief, about 200 to 500 ng PCR product was mixed with 50 ng pINIT vector, 1 × NEB CutSmart buffer, 1 µl BspQI (10 units) (NEB, isochizomer of SapI) and the volume was adjusted to 10 µl with water. The reaction was then incubated for 1 hour at 50 °C for BspQI DNA digestion, followed by 20 minutes at 80 °C for heat inactivation of BspQI. Afterwards, to the same reaction 1.25 µl 10 mM ATP and 1.25 µl T4 ligase (5 Weiss Units) (NEB) was added, and the reaction was incubated 1 hour at 25 °C for ligation. Subsequently, the ligase was heat inactivated 20 minutes at 65 °C, and 10 µl of the reaction was transformed into 50 µl chemically competent *E. coli* XL1-Blue cells. All ORFs carry an additional AGT codon at the 5′ end introducing a serine residue at the N-terminus, and an additional GCA codon at the 3′ end translating as C-terminal alanine residue. Subcloning into expression vectors was again performed according to the FX protocol as described^41^. In brief, about 500 ng pINIT vector (containing ORF) was mixed with 50 ng destination expression vector. The subsequent steps were identical to the cloning of ORFs described before, i.e. first digestion with BspQI followed by ligation with T4 ligase, and with subsequent transformation into XL1-Blue cells. Approximately 0.5–1.0 µg of the expression vector was electroporated into *M. smegmatis*, recovered for 3.5 hours at 37 °C in 7H9 and plated on 7H10 plates with suitable antibiotics. Clones carrying the expression vectors were confirmed by colony PCR.

### Small-scale protein production for in gel GFP analysis

10 ml 7H9 cultures supplemented with the suitable antibiotics were inoculated 1:25 with 7H9 precultures of *M. smegmatis* carrying pACE, pMEX or pMINT vectors and grown at 37 °C while shaking, until the cultures reached an OD_600_ of around 0.6. Cells were then induced with 100 ng/ml ATc (pMINT and pMEX) or 0.4% (w/v) acetamide (pACE) and grown overnight at 37 °C. 4 ml of cells were harvested in 2 ml tubes and resuspended in 350 μl 20 mM Tris/HCl pH 8, 200 mM NaCl. A spatula tip of acid-washed glass beads ≤106 µm (Sigma) was added and the cells were subsequently lysed with a FastPrep-24 Classic cell lysis machine (MP Biomedicals) for 3 cycles of 60 seconds with 6 m/s. The lysate was spun 15 minutes at 14’000 rpm at 4 °C with a F-45-30-15 rotor (Eppendorf) to pellet glass beads and cell debris. 40 μl of supernatant were mixed with 10 μl of 5 × SDS loading dye (120 mM TRIS pH 6.8, 50% glycerol, 100 mM DTT, 2% SDS (w/v), 0.1% bromophenol blue (w/v)), of which 10 µl were loaded on a 10% SDS-PAGE gel. The GFP-fusions were detected by *in gel* fluorescence with an ImageQuant LAS 4000 instrument (GE Healthcare). The exposure time was adjusted according to the intensity of the signal, and varied between 10 and 60 seconds.

### Large-scale protein production and purification for size-exclusion chromatography analysis

For large-scale protein production with *M. smegmatis*, 4.5 liter (MSMEG_6553/6554) or 9 liter (Rv1410) cultures of 7H9 supplemented with hygromycin were inoculated 1:25 with dense *M. smegmatis* precultures carrying the respective pACE constructs. Cells were grown at 37 °C until they reached an OD_600_ of approximately 0.6, and then were induced with 0.4% (w/v) acetamide. Protein production was induced overnight at 37 °C, and cells were harvested. All further steps were performed at 4 °C or on ice. Cells were resuspended in 20 mM Tris/HCl pH 8, 200 mM NaCl, and a spatula tip of DNase I was added and homogenized by sonication. Cells were lysed with a microfluidizer M-110P (Microfluidics) with 3 passes at 25 KPa. The lysate was first centrifuged for 30 minutes at 8’000 g to remove cell debris. The supernatant was then ultracentrifuged at 170’000 g for 2 hours to pellet the membrane vesicles. The vesicles were resuspended in 20 mM Tris/HCl pH 7.5, 150 mM NaCl (TBS) containing 10% glycerol, flash-frozen with liquid nitrogen and stored at −80 °C until further use. Membrane proteins were extracted and purified from the membranes essentially as described^25^. Briefly, membrane proteins were extracted with 1% (w/v) n-dodecyl-β-D-maltopyranoside (β-DDM) for 2 h, followed by ultracentrifugation at 170’000 g for 30 minutes. The supernatant containing extracted membrane proteins was loaded onto a Ni^2+^NTA Superflow gravity flow column (Qiagen). The column was washed with 30 column volumes with 50 mM imidazole pH 7.5, 150 mM NaCl and 10% glycerol, and the proteins were subsequently eluted with 5 column volumes of 200 mM imidazole pH 7.5, 150 mM NaCl and 10% glycerol. Eluted proteins were desalted in 20 mM Tris/HCl pH 7.5, 150 mM NaCl (TBS) using a PD-10 column (GE Healthcare), and the GFP-His_10_ tag was cleaved overnight by incubation with homemade 3C protease. Cleaved proteins were purified by reverse Ni^2+^NTA. Cleaved protein was washed from the column with 5 column volumes of 20 mM Tris/HCl pH 7.5, 150 mM NaCl (TBS). The proteins were concentrated using Amicon Ultra concentrators from Merck Millipore (30 kDa cutoff for Rv1410 and 100 kDa cutoff for MSMEG_6553/54) and loaded on a Superdex 200 Increase 10/300 GL (GE Healtcare) size-exclusion chromatography column.

### Production of pFLAG fusion proteins and Western blotting

*M. smegmatis* carrying chromosomally integrated pFLAG_attP_Rv1410 was grown in 7H9 medium with or without apramycin. 1 ml of a stationary phase culture were pelleted and resuspended in 500 µl 20 mM Tris/HCl pH 8, 200 mM NaCl. Cells were then lysed with a FastPrep-24 machine (for details see above), and a 2-fold dilution series of the supernatants was separated on a SDS-PAGE gel. Separated proteins were transferred to a membrane (Immobilon-PSQ, Merck). For this purpose, the gel and membrane were soaked in transfer buffer (2.9 g glycine, 5.8 g Tris, 0.1% SDS (w/v) and 20% methanol (v/v) in 1 liter), and the transfer was performed with a Trans-Blot® SD Semi-Dry Electrophoretic Transfer Cell (BIO-RAD) for 1 h at 12 V. The membrane was blocked in PBS buffer containing 5% (w/v) milk powder and 0.05% (v/v) Tween20 for 1 h at RT, incubated in blocking buffer with anti-FLAG antibody (Sigma, F3165) diluted 1:10’000 (v/v) for 1 h at RT while rotating and then washed 3x with washing buffer (PBS containing 0.05% (v/v) Tween20). An incubation of 1 h at RT while shaking in 30 ml of blocking buffer and α-mouse-HRP antibody (Sigma, A5278) diluted 1:30’000 (v/v) ensued. The membrane was washed again 3x with washing buffer, and finally, the proteins were visualized by adding Immobilon Western Chemiluminescent HRP Substrate (Merck) and measuring chemiluminescence using ImageQuant LAS 4000 (GE Healthcare).

### Testing the counterselection strength of pKO vectors

To test the counterselection strength of pKO vectors, *M. smegmatis* strains were generated with stably integrated pKO vectors via *attP*/*attB* site-specific recombination. For this purpose, the *attP* site was cloned via FX-cloning into the pKO vectors and the vectors were electroporated with the vector pMA_Int (see above) providing the integrase in *trans*^28^. Colonies of single clones were picked and inoculated in medium containing the respective antibiotic of the pKO vector, and the pKO integration site was checked by colony PCR. For assessing the counterselection strength, the strains were grown in liquid culture supplemented with the respective antibiotics to stationary phase (three nights). From this stationary phase culture 2 μl cells were streaked with a sterile loop onto gradient 7H10 plates devoid of antibiotics containing maximally 20% (w/v) sucrose and 0.2% (w/v) deoxy-galactose. The plates were incubated for three nights at 37 °C.

### Generation of gene deletions in *M. smegmatis* and *M. marinum* with pKO vectors

For the generation of gene deletions, the upstream and downstream flanking region of the targeted locus, each having a length in the range of 1 to 1.5 kb, were amplified from genomic DNA. The forward primers to amplify the upstream flanks and the reverse primers to amplify the downstream flanks were standard FX primers designed according to the FX primer tool (www.fxcloning.org). For reverse primers of the upstream flanking region, primers of the form 5′-tatatatGCTCTTCaAGG[…]-3′ were used, and for forward primers of the downstream flanking regions, primers of the form 5′-atatatGCTCTTCtCCT[…]-3′ were used, where […] corresponds to the sequence which anneals on the genomic DNA. Amplified flanking regions were then cloned in one restriction and ligation reaction into pINIT, sequenced, and then transferred by fragment exchange to the respective pKO vectors in *E. coli*. Approximately 1 µg of pKO vector was electroporated into 100 µl electrocompetent *M. smegmatis* or 400 µl electrocompetent *M. marinum* cells. Electrocompetent cells were prepared by established protocols^44^. Briefly, *M. smegmatis* or *M. marinum* cells were grown to approximately OD_600_ = 1, harvested and washed at least twice with 10% glycerol and taken up in 1:100 (*M. smegmatis*) or 1:10 (*M. marinum)* of the culture volume. For *M. smegmatis*, the procedure was performed on ice with ice-cold 10% glycerol. For *M. marinum* the 10% glycerol solution was supplemented with 0.05% (v/v) Tween 80 (Sigma) or Tyloxapol (Sigma) and preparation of the cells was performed at room temperature. For *M. smegmatis* the cells were recovered for 3.5 hours in 7H9 medium at 37 °C and then plated on 7H10 plates with the appropriate antibiotics. For *M. marinum*, the cells were recovered for 4 hours at 30 °C and the plates incubated at 30 °C. Colonies were picked and grown in 7H9 supplemented again with antibiotics. For both, *M. smegmatis* and *M. marinum*, clones were checked for successful integration by PCR. Primer pairs were designed in such a way, that one primer would anneal on the genome and the other on the integrated plasmid and yield a PCR product of the correct size only upon integration of the plasmid. The single cross-over transformants were subsequently grown in 7H9 without antibiotics to allow second recombination events to occur. Approximately 2 µl of cells from these cultures were then plated on 7H10 plates containing the counterselection substrates sucrose (20% w/v) and 2-deoxy-galactose (0.2% w/v). Clones were picked, grown in 7H9 and screened with colony PCR for gene deletions. Deletion mutants were in addition tested for antibiotic susceptibility by growing the strains with and without antibiotic supplementation in 7H9 medium. Positive clones were confirmed by repeating the PCR reaction using isolated genomic DNA as template. The resulting PCR product was finally sequenced to confirm the deleted region in the genomic context. A list of all primers used for cloning of the flanking regions can be found in Supplementary Table 3.

### MIC testing of *M. smegmatis* deletion mutants

*M. smegmatis* deletion mutants were grown for 3 nights at 37 °C in 7H9 medium while shaking. These cultures were diluted 1:15 (v/v) in fresh 7H9 medium and were grown again for 5 hours to obtain OD_600_ = 0.3 to 0.4. Subsequently, the culture was diluted 1:200 (v/v) to generate a working culture for an MIC test in a 96 well format, with 12 different concentrations of drugs per row present in a 2-fold dilution. For each MIC determination, two technical replicates were determined. Stock solutions of the drugs were prepared in H_2_O (ampicillin, streptomycin, vancomycin, kanamycin, ethidium bromide, ethambutol, isoniazid), 2.5% (v/v) DMSO (tetracyclin, erythromycin, rifampicin, novobiocin, chloramphenicol, pyrazinamide) or 0.1 M HCl (ofloxacin, ciprofloxacin). To prepare each well of the 96 well plate, 5 µl of drug at the appropriate concentration was mixed with 95 µl 7H9 medium and subsequently inoculated with 100 µl working culture. Plates were sealed with adhesive plate seals (Thermo Scientific) and incubated for 48 hours at 37 °C. 30 μl of 0.01% (w/v) resazurin was added into each well and the plate was again closed with a plate sealer and incubated for 6 h at 37 °C. The fluorescent intensity of metabolized resazurin (converted into resorufin) was determined with a Synergy H1 plate reader (BioTEK) at an excitation wavelength of 530 nm and emission wavelength of 590 nm. In the case of isoniazid, MIC values were determined by OD_600_ measurement since isoniazid interfered with fluorescence detection of resorufin.

### Generation and testing of the conditional knockout ideR in *M. smegmatis* with pCOND

The first 513 bp of the *ideR* gene encoding for MSMEG_2750 of *M. smegmatis* were amplified by PCR with the primers ideR Fw and ideR Rv (designed with www.fxcloning.org) and cloned via pINIT into the pCOND_Apr vector. Approximately 1 µg vector was electroporated into *M. smegmatis* and apramycin resistant clones were selected for integration with PCR, using the primers ideR_int Fw and ideR_int Rv. Additionally the presence of the *pip* promoter was confirmed with the primers Pip_Fw and Pip_Rv. One clone was selected and transformed with the integrative vector pFRA61.1 (obtained from Francesca Boldrin and Riccardo Manganelli, unpublished), containing the constitutively expressed Tet repressor TetR and the gene encoding the Pip repressor under the control of the P_*furA102*_ promoter controlled by TetR, as well as a streptomycin resistance marker. This clone was used for the subsequent CAS experiments for detection of soluble siderophores. For this purpose, cells of the conditional *ideR* mutant were grown in 100 ml minimal medium (6% glycerol (v/v), 5 g L-asparagine and 5 g KH_2_PO_4_ per 1 liter of medium, pH 6.8 adjusted with NaOH) supplemented with 1.9 μg/ml ZnSO_4_ · 7H_2_O, 0.4 μg/ml MnSO_4_ · 5H_2_O, 0.4 mg/ml MgSO_4_ · 7H_2_O, 25 μg/ml apramycin, 25 μg/ml streptomycin, and with different ATc concentrations. Soluble siderophore production was assessed every 24 hours by taking a 1 ml aliquot of the cultures and performing the CAS assay with the supernatant as described^39^.

### Data availability

All data generated or analyzed during this study are included in this published article. The vectors are made accessible to the community by depositing them on Addgene.

## Electronic supplementary material

Supplementary Information
