# Supplementary material for: A uniform cloning platform for mycobacterial genetics and protein production
Source: Sci Rep. 2018 Jun 22;8:9539. doi: 10.1038/s41598-018-27687-5 (PMC6015033; doi:10.1038/s41598-018-27687-5)
Supplement: Supplementary file 1 — Supplementary Information [file 41598_2018_27687_MOESM1_ESM.pdf]

SUPPLEMENTARY MATERIAL

A uniform cloning platform for mycobacterial genetics and protein production

Fabian M. Arnold[1], Michael Hohl[1,2], Sille Remm[1], Hendrik Koliwer-Brandl[1], Sophia Adenau[1], Sasitorn Chusri[1,3], Peter Sander[1], Hubert Hilbi[1] and Markus A. Seeger[1,*]

[1] Institute of Medical Microbiology, University of Zurich, Switzerland

[2] Current address: Department of Life Sciences, Imperial College London, UK

[3] Current address: Faculty of Traditional Thai Medicine, Prince of Songkla University, Thailand

* Corresponding author: m.seeger@imm.uzh.ch

Content:

Supplementary Figure S1

Supplementary Table S1

Supplementary Table S2

Supplementary Table S3

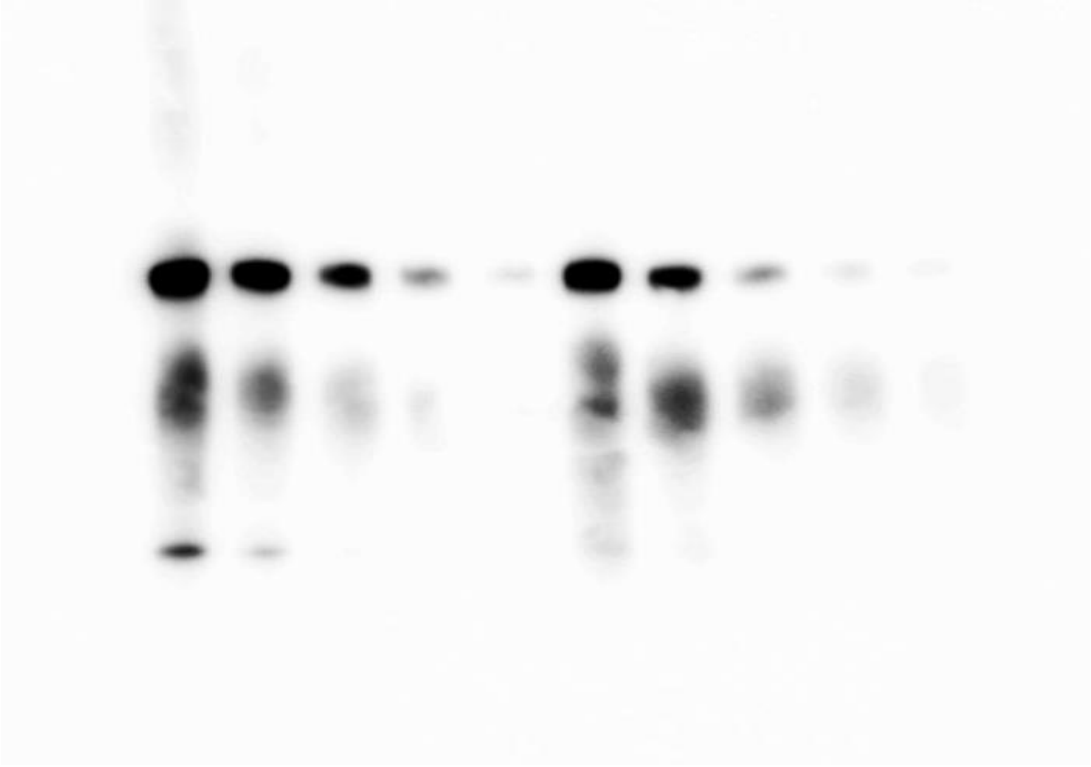

Supplementary Figure S1:

Original Western blot image used to generate Figure 4B.

Table S1: List of primers

**Primer name**	**Sequence (5' to 3')**	**5'-modification**
pGMCK Fw	TTATACATAGTTGCTTAAGCTGCAGGGG	Phosphorylation
pGMCK Rv	CATATGTATATCTCCTTCGCATGCCTCG	Phosphorylation
pBXC3GH Fw	AGTTGAAGAGCGACCTGCAGACTG	
pBXC3GH Rv	TTAATGATGGTGATGATGATGGTGATGATGATGAC	
pMINTC3H Fw	CATCATCATCACCATCATCATCACCATCAT	Phosphorylation
pMINTC3H Rv	TGGACCTTGAAACAAAACTTCTAATGCAGAAG	Phosphorylation
pBXNH3 Fw	CACCATCATCATCACCATCATCATCATC	
pBXNH3 Rv	TTATTATGCAGAAGAGCTGAACTAGTGG	
pMYC_OriM Fw	GTATtctagaGTGAGCCCACCAGCTCCGTAAG	
pMYC_OriM Rv	CAGTgaagactagcatGCCACGGATGCCACCACA	
pMINT_OriM Fw	GTATtctagaTTGATGCCTGGCAGTCGATCG	
pMINT_OriM Rv	CAGTgaagactagcatCCAGCTAGCAACAAAGCGACG	
pMINT/MEX_bb Fw	TCCCGTTGAATATGGCTCATAACACCC	
pMINT/MEX_bb Rv	TTGCAGTTTCATTTGATGCTCGATGAG	
pSET_apr Fw	gtaccatggAGGATCTTCACCTAGATCCTT	
pSET_apr Rv	gtatctagaATCAGCCGTCCAAATGCGGG	
pMYC_hyg Fw	gtatctagaCTAGCGTACGATCGACTGCCAG	
pMYC_hyg Rv	gtaccatggATAGTTGCCT-GACTCCCCGACGTG	
pMYC_bb Fw	TAACAGCTTATCGATGTCGACGTAGTTAACTAGCG	Phosphorylation
pMYC_bb Rv	CATGGTGGACTCCCTTTCTCTTATCGGG	Phosphorylation
pACE_sapI Fw	GTGGCGTCGGGCTCaTCTACGTGGGTG	
pACE_sapI Rv	CACCCACGTAGAtGAGCCCGACGCCAC	
pACE_Apr Fw	GCCACGTCGGGGAGTCAGGCAACTA	Phosphorylation
pACE_Apr Rv	CTCTAGCTGATCACCGCGGCCATGA	Phosphorylation
pMINTΔTetR Fw	ACCAGGCCTAGATCTGGGGACCCTAG	Phosphorylation
pMINTΔTetR Rv	GGATCATCGAGCCGAGAACGTTATCG	Phosphorylation
pBXCF3GH Fw	CATGATATCGATTACAAGGATGACGATGACAAGTTAGAAGTTTTGTTTCAAGGTCCAC	Phosphorylation
pBXCF3GH Rv	ATCTTTATAA-TCACCGTCATGGTCTTTGTAGTCTGCAGAAGAGCAGAACTAGTGG	Phosphorylation
pMINTΔTetR_bb Fw	CTTGTTGTCGTAGGTCTGCAGCGC	
pMINTΔTetR_bb Rv	GCCAGTCTGCAGGTCGCTCTTC	
pBXC3F3GH_bb Fw	GAAGAGCGACCTGCAGACTGGC	
pBXC3F3GH_bb Rv	CTTGTCATCGTCATCCTTGTAATCG	
pCOND_APR_bb Fw	TAGTGAGCATTCCGCTTCCTCGCTCACTG	Phosphorylation
pCOND_APR_bb Rv	ATCCTCGTGTGGTTGCTAGATGGC	Phosphorylation

pMA_FX Fw	ctgctcttcagcaTCACTGCCCGCTTTCCAGTC	Phosphorylation
pMA_FX Rv	atgctcttcaactTATTACGTCGCGCTCACTGGC	Phosphorylation
Int Fw	atatatGCTCTTCtAGTTTGTTGCTAGCTGGCGGGAAC	
Int Rv	tatataGCTCTTCaTGCATATGGGCCGGTCAAGATAGGT	
pMA_SapI Fw	TCCCATTCGCCATTCAGGCT	Phosphorylation
pMA_SapI Rv	ATCCCTTAACGTGAGTTTTCGT	Phosphorylation
pDB77 Fw	TAAGTTGGGTAACGCCAGGG	
pDB77 Rv	GGTTTTTACCCCCTCTCGGC	
pMA_DB77 Fw	TGATCCCGGAAGAaCTGGTG	
pMA_DB77 Rv	CACCAGtTCTTCCGGGATCA	
pKOΔsacB Fw	GTCGtctagaTGAATCGCCCCATCATCCAGC	
pKOΔsacB Rv	GTATggcgcgccTCCCATTCGCCATTCAGGCT	
pINIT_sacB Fw	GTATggcgcgccCTGATGCCGCATAGTTAAGCCAG	
pINIT_sacB Rv	TCCTACTCAGGAGAGCGTTCACC	
pTE_bb Fw	ATCAACGAGGACAGTCGCACGAC	Phosphorylation
pTE_bb Rv	AGGCTCGCGTAGGAATCATCCG	Phosphorylation
pTE Fw	atatatGGATCCgctcttcagcaTTCCGCTTCCTCGCTCACTGAC	
pTE Rv	atatatGGATCCTCGTGTGGTTGCTAGATGGCTC	
pFRA50 Fw	ATATATggatccAGATCTCCATCCTGACGGATGG	
pFRA50 Rv	ATATATggatccGCTCTTCAACTCATTCAGGCTCCTTGTACGGTG	
ideR Fw	atatatGCTCTTCtAGTAACGATCTTGTCGATACCACCGAG	
ideR Rv	tatataGCTCTTCaTGCTTCGGTCAGCTGGCGGACCACCAC	
ideR_int Fw	ATATATggatccAGATCTCCATCCTGACGGATGG	
ideR_int Rv	TCAGACCTTCTCGACCTTGACG	
Pip Fw	ACGTCGACACCAAGGACCAG	
Pip Rv	TCAGGCCTGTTCGACCATCG	

Table S2: List of plasmids used for plasmid construction

Plasmid name	Description	Resistance	Reference
pINIT	Initial sequencing vector for FX-cloning	Chl	24
pBXC3GH	FX expression vector based on the arabinose promoter with C-terminal GFP and His_{10} tags	Amp	24
pBXNH3	FX expression vector based on the arabinose promoter with N-terminal His_{10} tag.	Amp	24
pGMCKq1-10M1-sspBmyc	TetR based expression vector. Used as backbone for the pMINT and pMEX vectors.	Kan	6
pSET152	Integrative vector used to amplify apramycin resistance gene	Apr	42
pMYC	Acetamide inducible expression vector for mycobacteria. Used as backbone for the pACE vectors.	Hyg	Addgene ID 42192
pDB77	Suicide vector for knockout generation in mycobacteria, harbouring the *galK* counterselection gene. Used as backbone for the pKO vectors.	Kan	29
pTE	Replicative vector for mycobacteria used as backbone for pCOND vectors. Two version with apramycin or hygromycin resistance.	Apr / Hyg	Addgene ID 20320

Table S3: List of primers used to clone mycobacterial flanking regions

MSMEG_5008/5009	upstream flank Fw	ATATATGCTCTTCTAGTCGCGCAACTCGTGACCTTCCACGTACC
	upstream flank Rv	TATATATGCTCTTCAAGGCCCGCTACTCTCGCACACGATCCTGAG
	downstream flank Fw	ATATATGCTCTTCTCCTGAAACTGCTGGTGCTCGACGAGGCCAC
	downstream flank Rv	TATATAGCTCTTCATGCGCGCAGCATCTTGCGGTTCTGGTTGGC
MSMEG_5659/5660	upstream flank Fw	ATATATGCTCTTCTAGTTACACCCGCGAGCTCGCCGAGTACTG
	upstream flank Rv	TATATATGCTCTTCAAGGAGCGAGCGCATGGTGCAACGTGCACTG
	downstream flank Fw	ATATATGCTCTTCTCCTTCACGCAGCGCTCGCATCGGTGTCGAA
	downstream flank Rv	TATATAGCTCTTCATGCCGCCACGCTGGTCAAACTGTTCGGCAG
MSMEG_6225	upstream flank Fw	ATATATGCTCTTCTAGTTCTACGTGGTCGGTGTGCTGCTGTG
	upstream flank Rv	TATATATGCTCTTCAAGGTCCTGTACGGAAAAAAGGGGTGTG
	downstream flank Fw	ATATATGCTCTTCTCCTTTCTACCGCAGCAACGTCGACGTG
	downstream flank Rv	TATATAGCTCTTCATGCACGAACTCACCGACTACGACCTGC
MSMEG_3563	upstream flank Fw	ATATATGCTCTTCTAGTGACTCGCCTTCCTCGGTCATGC
	upstream flank Rv	TATATATGCTCTTCAAGGACCTCACCTGGCACCGCGAAC
	downstream flank Fw	ATATATGCTCTTCTCCTGACGTCCTTTCACGCATCCGGC
	downstream flank Rv	TATATAGCTCTTCATGCCCTTCGATGGTCATGATGTGCGCC
MSMEG_5187	upstream flank Fw	ATATATGCTCTTCTAGTAAGGTGCACAGATGACTTCCACCG
	upstream flank Rv	TATATATGCTCTTCAAGGCGGTTCTTCGATGTCATGTTGCGTAC
	downstream flank Fw	ATATATGCTCTTCTCCTCCTGGACTTCTTCGTATCGCTGGCG
	downstream flank Rv	TATATAGCTCTTCATGCTCGAACCCCATGGATCCCATCTCG
MSMEG_6390	upstream flank Fw	ATATATGCTCTTCTAGTGCTGGTGGTACGTGTCGAACTTCGG
	upstream flank Rv	TATATATGCTCTTCAAGGCGCCCCAATGGTATGTCAATAAGTC
	downstream flank Fw	ATATATGCTCTTCTCCTATCGGCTGGATCTGCACGCACG
	downstream flank Rv	TATATAGCTCTTCATGCTTCAGCCACGACCAGACGGTGTTCC
MSMEG_5075_5076	upstream flank Fw	ATATATGCTCTTCTAGTACTGAACTTTCGCTGAGAAACCCGACC
	upstream flank Rv	TATATATGCTCTTCAAGGTCATGTCGCCTCCTCCGAGGTGC
	downstream flank Fw	ATATATGCTCTTCTCCTGGTGACAGCGCTGTTCGTGATGGC
	downstream flank Rv	TATATAGCTCTTCATGCCACGCGATTCTCGACAAGAACGTCG
MSMEG_1502_1503_1504	upstream flank Fw	ATATATGCTCTTCTAGTGAAGGCACCAGCATCGAACAGATCGC
	upstream flank Rv	TATATATGCTCTTCAAGGTACCGGCGGATTCACACTGCTCGGG
	downstream flank Fw	ATATATGCTCTTCTCCTGTTCATCGATTCGGCGTGGAACCTG
	downstream flank Rv	TATATAGCTCTTCATGCTCAAGGAGATGGACCAGCTGCTCTC
MSMEG_6509_6510	upstream flank Fw	ATATATGCTCTTCTAGTTCGCGTCGATCAACTTCGTCATCGC
	upstream flank Rv	TATATATGCTCTTCAAGGTCATGAGTCCCTTTCCTGCAGCAGC
	downstream flank Fw	ATATATGCTCTTCTCCTTGGTCCGATAACCGACCAACCAACTG
	downstream flank Rv	TATATAGCTCTTCATGCCCGATCTCGTTGAGCACCACCATG
MSMEG_00017/0018	upstream flank Fw	ATATATGCTCTTCTAGTTCGTGATGTCGGCCGATCTGGTGC
	upstream flank Rv	TATATATGCTCTTCAAGGTCACGCCAAAGTGGCCGTATTGTCG
	downstream flank Fw	ATATATGCTCTTCTCCTGTCGAAAACGGCACGCATGCAGAG
	downstream flank Rv	TATATAGCTCTTCATGCTCGGCCAGTTGCGACTCGATCTCC

MSMEG_1642	upstream flank Fw	ATATATGCTCTTCAAGTGAAGCGCCCGTGGAAACTCT
	upstream flank Rv	TATATATGCTCTTCAAGGCAAAACTGCGCCTTTTCACG
	downstream flank Fw	ATATATGCTCTTCACCTGCGGTCAACTCTATCGGTAC
	downstream flank Rv	TATATAGCTCTTCATGCACGAAGCCGGAGTCGAACGAC
MSMEG_4380	upstream flank Fw	ATATATGCTCTTCAAGTGACGACGTGTTGCTGCGAGA
	upstream flank Rv	TATATATGCTCTTCAAGGGACCGACTCCTGAGTTTGCC
	downstream flank Fw	ATATATGCTCTTCACCTGATCATGGACCACATCACCT
	downstream flank Rv	TATATAGCTCTTCATGCCCTGGGTGTAGTGGATGATC
MSMEG_2631	upstream flank Fw	ATATATGCTCTTCTAGTCGAAACGTCTCGCCAAGCGGATC
	upstream flank Rv	TATATATGCTCTTCAAGGATCAGCCAAGGGCCCGTGCG
	downstream flank Fw	ATATATGCTCTTCTCCTTGACGAGGTCAGCGCTTGATCG
	downstream flank Rv	TATATAGCTCTTCATGCGTCACCGTCACGTTCACCTCGAAAC
MSMEG_3815	upstream flank Fw	ATATATGCTCTTCTAGTCGATTTCGCGCTACTTCCTCAGATACG
	upstream flank Rv	TATATATGCTCTTCAAGGCGCTGAGTAAGTGAGTGAGTGACTGAG
	downstream flank Fw	ATATATGCTCTTCTCCTTCATTTCAGCCCGGCGGCATC
	downstream flank Rv	TATATAGCTCTTCATGCGATCATCCCGTCGTACGAGGAAC
MSMEG_6221	upstream flank Fw	ATATATGCTCTTCTAGTCAACTACGTCACGCCCGACGAC
	upstream flank Rv	TATATATGCTCTTCAAGGCGGCTCAGAGCTTTCCGGTG
	downstream flank Fw	ATATATGCTCTTCTCCTTCGGCCGAGCTTAATCGCCTAACG
	downstream flank Rv	TATATAGCTCTTCATGCGCCCACCCTGGGGGATATTC
MSMEG_3705	upstream flank Fw	ATATATGCTCTTCTAGTGGACTTCAGCGCTGCGACGATC
	upstream flank Rv	TATATATGCTCTTCAAGGCAGCGTCTCCTCGTCGGGATCAG
	downstream flank Fw	ATATATGCTCTTCTCCTCTCGCTGAGTTCTGCGCTCG
	downstream flank Rv	TATATAGCTCTTCATGCCAGATCGGCGGTCTTGGTCTCCATC
MSMEG_3670	upstream flank Fw	ATATATGCTCTTCTAGTTGAGCTCACGGCACGGCTGTAC
	upstream flank Rv	TATATATGCTCTTCAAGGCCGCACCGCAACAACCTAGC
	downstream flank Fw	ATATATGCTCTTCTCCTTTCGGATCCCACGAACGCGAGGCC
	downstream flank Rv	TATATAGCTCTTCATGCAGTGGTGACAGAACAGCGTGACC
MSMEG_3672	upstream flank Fw	ATATATGCTCTTCTAGTAAGTACGTGCACCTGGGCAACCACAC
	upstream flank Rv	TATATATGCTCTTCAAGGTCACGGTCGGGCCTCGCGTTCGTG
	downstream flank Fw	ATATATGCTCTTCTCCTCCTCAGGTGTGTTCACGATCGAGTTG
	downstream flank Rv	TATATAGCTCTTCATGCAGTGGTGACAGAACAGCGTGACC
MS_5046	upstream flank Fw	ATATATGCTCTTCTAGTAAGTACGCCGCGGTGATCGT
	upstream flank Rv	TATATATGCTCTTCAAGGACCCTCGCTCACTGAGCGAACAG
	downstream flank Fw	ATATATGCTCTTCTCCTATGACAAGTCGGTGGTGAATCGGG
	downstream flank Rv	TATATAGCTCTTCATGCGTTAACGAACGGAGATTCCCGCGCGT
MS_5670	upstream flank Fw	ATATATGCTCTTCTAGTTGAGATTGTGTTGTCCGCTGATGAGCG
	upstream flank Rv	TATATATGCTCTTCAAGGTGAACTCGCCCAGGACACG
	downstream flank Fw	ATATATGCTCTTCTCCTCATACAACGATTGTGAAAGCACAACTG
	downstream flank Rv	TATATAGCTCTTCATGCCTTCGACGGTTTCCCGCGCAAC
